# Telomeres and Telomerase in Cardiovascular Diseases

**DOI:** 10.3390/genes7090058

**Published:** 2016-09-01

**Authors:** Jih-Kai Yeh, Chao-Yung Wang

**Affiliations:** 1 Department of Cardiology, Chang Gung Memorial Hospital, 33305 Taoyuan, Taiwan; potential.ya@gmail.com; 2 Chang Gung University College of Medicine, 33302 Taoyuan, Taiwan

**Keywords:** telomere and telomerase, cell senescence, cardiovascular diseases

## Abstract

Telomeres are tandem repeat DNA sequences present at the ends of each eukaryotic chromosome to stabilize the genome structure integrity. Telomere lengths progressively shorten with each cell division. Inflammation and oxidative stress, which are implicated as major mechanisms underlying cardiovascular diseases, increase the rate of telomere shortening and lead to cellular senescence. In clinical studies, cardiovascular risk factors such as smoking, obesity, sedentary lifestyle, and hypertension have been associated with short leukocyte telomere length. In addition, low telomerase activity and short leukocyte telomere length have been observed in atherosclerotic plaque and associated with plaque instability, thus stroke or acute myocardial infarction. The aging myocardium with telomere shortening and accumulation of senescent cells limits the tissue regenerative capacity, contributing to systolic or diastolic heart failure. In addition, patients with ion-channel defects might have genetic imbalance caused by oxidative stress-related accelerated telomere shortening, which may subsequently cause sudden cardiac death. Telomere length can serve as a marker for the biological status of previous cell divisions and DNA damage with inflammation and oxidative stress. It can be integrated into current risk prediction and stratification models for cardiovascular diseases and can be used in precise personalized treatments. In this review, we summarize the current understanding of telomeres and telomerase in the aging process and their association with cardiovascular diseases. In addition, we discuss therapeutic interventions targeting the telomere system in cardiovascular disease treatments.

## 1. Introduction

Aging is a major risk factor for cardiovascular diseases (CVDs), including myocardial infarction (MI), stroke, hypertension, and chronic heart failure (HF). The prevalence of CVDs significantly increases with age, and CVDs are a major cause of chronic disability and the leading cause of death worldwide. Although the prevention and treatment of CVDs have substantially progressed during the previous two decades, morbidity and mortality rates associated with CVDs remain high and impose a tremendous burden on the national healthcare system. According to heart disease and stroke statistics, CVDs account for 30.8% of deaths [1]; that is, approximately one of every three deaths in the United States is caused by CVDs. Moreover, only 20% of people who died of CVDs in 2011 were aged less than 65 years and 34% were aged less than 75 years. As the global population is aging, the number of older people (aged ≥60 years) will exceed that of younger people (aged <15 years), and the proportion of older people will reach 21% by 2050 [2]. This indicates that CVDs in the older population are a crucial healthcare challenge that must be addressed. A superior understanding of the complex interaction between the aging process and CVDs is required to develop a novel therapeutic target for older patients.

Several epidemiologic surveys have reported an association of short telomere length (TL) with CVD [3,4,5,6] and cardiovascular mortality [7,8]. For instance, the Cardiovascular Health Study reported that each shortened kilobase pair of TL corresponded to a threefold increased risk of MI and stroke [3]. A recent systematic review and meta-analysis reported a constant positive association of decreased leukocyte TL (LTL) with cardiometabolic outcomes, where one standard deviation (SD) decrease in LTL was significantly associated with a 21%, 24%, and 37% increased risk of stroke, MI, and type 2 diabetes mellitus, respectively [5]. In this review, we discuss and review the current knowledge of the role of telomeres in CVDs.

## 2. Telomeres and Telomerase

In humans, telomeres consist of hundreds to thousands of repetitive sequences of TTAGGG at chromosomal ends for maintaining genomic integrity [9]. Because the DNA replication is asymmetric along double strands, a sequence at the 3′-hydroxyl end would lose 30–200 nucleotides with each DNA replication and cell division [10]. Telomeres provide a repetitive noncoding sequence at the 3′ end to prevent the loss of critical genetically encoded information during replication. Moreover, telomeres are coated with a complex of six capping proteins (telomere repeat-binding factor 1 (TRF1), telomere repeat-binding factor 2 (TRF2), repressor activator protein 1 (Rap1), TRF1 and TRF2 interacting nuclear protein 2 (TIN2), Tripeptidyl-peptidase 1 (TPP1), and protection of telomere 1 (POT1)), also known as shelterin proteins [11], which are packed into a compact T-loop structure to prevent the DNA repair machinery from mistaking telomeres for double-stranded DNA breaks. Therefore, TL have been proposed as a mitotic clock that measures how many times a cell has divided.

The human telomerase is responsible for maintaining and elongating TL and consists of the telomerase RNA component (TERC) and telomerase reverse transcriptase (TERT), the catalytic component. The TERT uses the TERC as a template to synthesize new telomeric DNA repeats at a single-stranded overhang to maintain TL. Some cells such as germ cells, stem cells, hematopoietic progenitor cells, activated lymphocytes, and most cancer cells have a high level of telomerase activity to overcome telomere shortening and maintain limitless cell division. However, somatic cells generally have a low or undetectable level of telomerase activity with limited longevity. The TL and integrity are regulated through the interplay between the telomerase and shelterin proteins [12]. Telomerase activity decreases with age but increases markedly in response to injury [13]. In the mammalian heart, telomerase expression is low but functionally significant. A substantial increase in telomerase expression was detected in cardiomyocytes, endothelial cells and fibroblasts of cryoinjured adult mice hearts, which implies that telomerase plays a role in regulating tissue repair and regenerative [14].

When a telomere is shortened to a critical length, the cell enters cellular senescence, which initiates a series of changes in the gene expression of replicative cell-cycle inhibitors and inhibits proliferation and then finally into apoptosis [15] as known as replicative senescence. By contrast, stress-induced premature senescence (SIPS) is triggered by external stimuli, including oxidizing agents and radiation, leads to the premature activation of the cellular senescence process not associated with telomere shortening. The senescent cells alter their morphology and secretary phenotype in autocrine and paracrine patterns. This active altered secretion pattern has been termed as the senescence-associated secretory phenotype (SASP). They secrete IL-6 and IL-8, intercellular adhesion molecule 1 (ICAM-1), metalloproteases, monocyte attractants, plasminogen activator inhibitor 1, and vascular endothelial growth factor [16,17]. Senescent cells contribute to inflammation and promote apoptosis, tissue remodeling, and repair through their SASP. Hence, chronic inflammation initiates a vicious cycle that enhances telomere dysfunction and the accumulation of senescent cells. Cell senescence aggravates chronic inflammation and accelerates aging and the development of aging-associated diseases [17].

## 3. Implication of Experimental Studies

Atherosclerosis is developed through a complex multifactorial process and contributes to major CVDs [18]. Endothelial dysfunction and damage by stimuli are usually a starting point with an increased expression of adhesion molecules. It promotes circulating leukocyte attachment and transendothelial migration. Then, activated monocytes transform into macrophages and uptake oxidative lipoproteins to become foamy cells, which are the inner core of fatty streaks. Furthermore, numerous inflammatory cytokines produced by activated leukocytes promote the proliferation and migration of vascular smooth muscle cells (VSMCs). They synthesize collagen enclosing the growing lipid core with the fibrous cap. The stability of the atherosclerotic plaque depends on the thickness of the fibrous cap and the degree of inflammation. There is growing evidence about telomere biology and cell senescence involving in the pathogenesis of cardiovascular diseases. For example, hemodynamic shear wall stress in some anatomical areas, associated with increased cell turnover and a higher telomere attrition rate, make the vascular tissue susceptible to atherosclerosis [19,20]. Shorter telomeres have been observed in endothelial cells and VSMCs of the atherosclerotic arterial wall, which is consistent with various regions of the human vasculature [19,20,21]. We review current understanding of the role of telomeres and telomerase in the functional regulation of vascular cells afterwards.

### 3.1. Endothelial Cells 

The functional integrity of endothelial cells is essential for vascular health and preventing the development of atherosclerosis. Substantial evidence indicated that a biological relationship is present among shortened TL, cellular senescence, and atherosclerosis [22]. Molecular mechanisms, such as an increases in reactive oxygen species (ROS), deceased nitric oxide bioavailability, and reduced TERT activity, have been found to be associated with the process of aging and atherogenesis [23,24,25]. Dysregulation of the redox balance and TERT appear to accelerate the process of the senescence of vascular endothelial cells, which are found in the atherosclerotic regions of human coronary arteries [24].

Telomerase activity is repressed in endothelial cells freshly isolated from intact endothelia, as in most somatic cells with low proliferative activity. However, telomerase activity is present in cultured endothelial cells and has been shown to be reversibly upregulated by fibroblast growth factor-2 (FGF-2) [26] and nitric oxide [27]. In additional, the serine/threonine kinase Akt have been implicated in the activation of TERT by posttranscriptional modification (phosphorylation) [28,29]. This suggests Akt is a potential therapeutic target for induction of telomerase activity in injured endothelia by mitogens, which enhance tissue repair and regenerative processes after vascular events.

### 3.2. Vascular Smooth Muscle Cells

In human atherosclerosis study [22], VSMCs in plaque exhibit oxidative DNA damage and increased expression of senescence markers such as senescence-associated β-galactosidase, cyclin-dependent kinase inhibitors p16 and p21, decreased expression of cyclin D and cyclin E, and hypophosphorylation of the retinoblastoma protein. VSMCs in plaques demonstrated markedly shorter telomeres, which closely are correlated with severity of atherosclerosis. The senescent VSMCs in atherosclerotic plaque also exhibit limited capacity of proliferation with increased activity of matrix-degrading enzymes as well as promote the thinning of fibrous caps and plaque rupture, which may lead to subsequent thrombosis, MI, or stroke [30]. These observations indicate that telomere attrition and premature cellular senescence by oxidant stress play an important role in pathogenesis of human atherosclerosis.

Regarding the role of telomerase in the proliferation of VSMCs, Minamino et al. demonstrated that VSMC proliferation is closely correlated with increased telomerase activity and that the protein kinase inhibitor H7 suppresses the activation of telomerase in the cytoplasm and nucleus, as well as reducing the growth of VSMCs [31]. They propose a mechanistic model to link the activation of telomerase and proliferation of VSMCs, in which TERT is activated by phosphorylation in the cytoplasm, followed by nuclear translocation during cell growth. As implied by the evidence derived from endothelial cells, the activation of TERT by posttranslational modification can extend cell lifespan and proliferative capacity, offering a regulatory mechanism through which to manipulate telomere attrition and cellular senescence in aging-associated diseases.

### 3.3. Immune Cells

Inflammation is believed to play a crucial role in atherosclerosis. The presence of short LTL in human atherosclerosis can be mainly attributed to the increased leukocyte consumption in inflammatory processes and accelerated telomere loss per replication in enhanced oxidative stress. Similarity, short TL in T lymphocytes are also exhibited in patients with chronic infection [32] and inflammatory disease [33] in conjunction with accelerated leukocyte turnover. Despite this, lymphocytes can transiently express telomerase during development and clonal proliferation to maintain TL. A progressive reduction in transiently enhanced telomerase activity was observed during successive stimulations and was accompanied by telomere loss [34].

For example, chronic cytomegalovirus (CMV) infection was reported to be associated with atherosclerosis in humans [35], which might be explained by an accumulation of senescent CMV specific CD8^+^ T cells and associated immune responses to acute inflammation. Spyridopoulos et al. reported TL in granulocytes, monocytes, peripheral blood stem cells and progenitor cells was consistently 500 bases shorter in leukocytes from patients with CAD than in control subjects. However, only cytotoxic CD8^+^ T cells in CMV-seropositive CAD patients were found to have increased TL loss of 1000 bases than in control subjects [36]. In their further prospective investigation, they confirmed acute and persistent depletion of terminally differentiated CD8^+^ T cells in CMV-seropositive patients through programmed cell death-1-dependent apoptosis processes during acute myocardial infarction [37]. Furthermore, an intensified immune response after myocardial ischemia and perfusion was observed in CMV-seropositive patients, which might augment cytotoxic damage in the ischemic myocardium and result in larger infarct area and ventricular dysfunction [38]. Different responses to acute myocardial infarction in CMV-seropositive and seronegative subjects were found in the studies, which the authors hypothesized that a higher portion of activated late differentiated CD8^+^ T cells left blood circulation and migrated into the target organ during acute inflammation in CMV-seropositive patients. The activated CD8^+^ T cells exhibit cytotoxic damage in the target organ and then were depleted spontaneously via an apoptosis mechanism. Homeostatic proliferation of T cells after acute stage accelerated TL shortening of CD8^+^ T cells and immunosenescence in CMV-seropositive patients. It could serve as a model of further investigations about the regulatory mechanisms of leukocytes telomere shortening in circumstances of acute inflammation.Telomere loss is a marker of leukocyte senescence. Immunosenescence is generally used to described the age-associated functional decline of immune system [39], which is associated with increased susceptibility to infectious diseases, reduced immunity from vaccination, increased autoimmunity, and tissue damage from dysregulated inflammation. This process is characterized by the presence of high proportions of CD8^+^ CD28^−^ T cells with features of replicative senescence, such as shortened telomeres, loss of telomerase activity, and enhanced secretion of inflammatory cytokines [40]. Accumulation of these cells restricts the development of new functional cells that are specific for other antigens, thus compromising overall immunity. Moreover, low-grade systemic inflammation through secretion of TNFα and IL-6 by these CD8^+^ T cells is believed to be a causative factor in numerous age-related conditions, including atherosclerosis, cardiovascular diseases, metabolic syndrome, obesity, type 2 diabetes, osteoporosis, and osteoarthritis [41]. In animal models, chronic progressive low-grade inflammation through ROS-mediated DNA damage in nfkb1^−/−^ mice, which entails loss of repressive regulation of proinflammatory gene transcription, results in premature aging with telomere dysfunction, cell senescence, and impaired tissue regeneration in multiple organ systems. This phenomenon can be reversed by anti-inflammatory or antioxidant treatment [42].

However, in TERC^−/−^ mice model, generation four TERC^−/−^ ApoE^−/−^ mice developed fewer atherosclerotic lesions compared with generation four TERC^+/+^ ApoE^−/−^ mice, which implies that the absence of telomerase activity is protective for atherosclerotic disease. This conflicting result might be explained by replicative senescence in immune cells. In this study, the proliferative capacity of macrophages and lymphocytes was decreased in generation four TERC^−/−^ ApoE^−/−^ mice compared with generation four TERC^+/+^ ApoE^−/−^ [43]. In addition, later generation TERC^−/−^ mice have been observed to have more severe structural defects than earlier generation TERC^−/−^ mice, including spleen atrophy and bone marrow proliferative defects, which limits differentiation and proliferation of functional immunocompetent cells and thus restricts atheroma progression. Therefore, telomere attrition might consistently cause cellular senescence, and apoptosis in difference cell types, but have variable effects on complex disease processes, such as atherogenesis or tumorigenesis in tissues or species and disease-specific patterns.

### 3.4. Cardiomyocytes 

An experimental study reported that telomerase knockout mice (TERC^−/−^) have progressively shortened telomeres in the later generation along with attenuation in myocyte proliferation and an increase in apoptosis [44]. In addition, these mice exhibit ventricular dilation, thinning of the wall, and cardiac dysfunction, mimicking the end-stage dilated cardiomyopathy in humans. By contrary, forced expression of TERT in the cardiac muscle of mice promotes cell proliferation, hypertrophy, and survival [45]. Furthermore, enhanced telomerase activity in insulin-like growth factor-1 transgenic mice has been shown to delay cellular aging and promote cell growth, thus preventing ventricular dysfunction [46]. In humans, endomyocardial biopsies from patients with HF reveal shortened telomeres, increased cellular senescence, and cell death [47]. Telomere dysfunction and increased susceptibility to apoptosis in cardiac myocytes is thought to be underlying mechanism of HF. The hypothesis has tested in cultured human cardiomyocytes, which defective expression of TRF2, a telomere end-capping protein, triggered telomere erosion, activation of the DNA damage checkpoint kinase, Chk2 and apoptosis [48].

Several studies have indicated that the heart undergoes the regeneration of some cardiac myocytes throughout life [49,50]. However, the proliferative and regenerative potential of cardiac progenitor cells partially depend on the integrity of telomeres and activity of telomerase. Stem cells in young cardiac myocytes have active telomerase and stable TL, whereas stem cells in the aging heart exhibit telomere attrition and express cell senescence markers. Hence, the decrease in the cardiac myocyte regeneration potential and accumulation of old dying cells finally lead to cardiac pumping failure. These findings indicate that telomere biology play an important role in regulation of regenerative capacity in myocardium and involved in the pathophysiology of HF.

### 3.5. Endothelial Progenitor Cells

The repair mechanisms for vascular atherosclerosis are dependent on endothelial progenitor cells (EPCs), which originate from hematopoietic stem cells (HSCs) in the bone marrow [51]. The shortening of the TL of HSCs caused by inflammation or oxidative stress limits the number and function of EPCs and impairs the replicative potential in the injured part of the vasculature [52,53]. However, EPCs with human TERT transduction have enhanced mitogenic and migratory activity in cell cultures and improved neovascularization in murine model of hindlimb ischemia. These findings demonstrate that EPCs with enhanced telomerase activity could be a novel therapeutic strategy for patients with severe ischemia heart disease and post infarct cardiomyopathy [54].

Furthermore, we observed mutations in circadian gene Per2 caused vascular senescence and impaired impairs ischemia-induced revascularization through the alteration of EPC function [55], which may explained the clinical observation of the link of alteration of the circadian and cardiovascular diseases.

## 4. Implications for Cardiovascular Diseases

### 4.1. Measurement of Telomere Length

**Figure 1:**
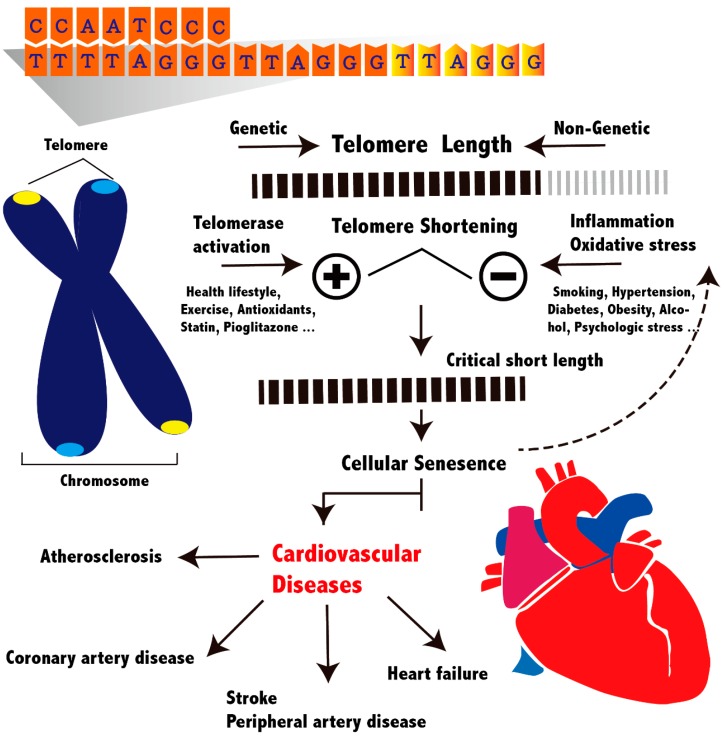
Schematic overview of telomere length and cardiovascular diseases. Individual variations of telomere length are affected by genetic and non-genetic factors. Critically short telomeres lead to cellular senescence and dysfunction, which contribute to atherogenesis and reduce repair and regenerative capacity in cardiovascular system. Disease promoting factors, such as smoking and hypertension, accelerate telomere shortening through inflammation or increased oxidant stress. However, disease protective factors, such as exercise and statin use, can activate telomerase activity and maintain telomere length.

Peripheral leukocyte DNA has been most commonly used in epidemiological studies to measure TL because a blood sample can be easily obtained. A consistent synchrony exists between LTL and somatic cells, including vascular cells, within people [56]. The two methods most commonly used in clinical studies are Southern blotting and quantitative polymerase chain reaction (qPCR). Southern blotting has an advantage of measuring the absolute LTL, including the proportion of very short telomeres. Cells with very short TL are closely associated with cellular senescence, regardless of mean TL, because only one critically short telomere can force a cell to enter senescence [57,58]. However, Southern blotting requires numerous DNA samples (2–3 µg per assay) and is time-consuming and expensive. Thus, qPCR is used in most epidemiological studies. The fundamental difference in laboratory methods among individual studies might contribute to controversial results. Recently, a study compared these laboratory methods performed in two independent laboratories to measure the same samples. Both the q-PCR and Southern blotting provided highly reproducible and correlated results [59]. The overview of TL and associated cardiovascular diseases is depicted in Figure 1.

### 4.2. Genetic Factors

TL is largely inherited [60,61] and is modulated by several intrinsic and environmental factors throughout life [62]. Rare mutations in genes that maintain and regulate TL have been identified in monogenic catastrophic diseases with premature tissue degeneration and organ dysfunction, such as dyskeratosis congenita, idiopathic pulmonary fibrosis, and aplastic anemia. Extensive inter-individual variations in LTL in the general population may attributed to single-nucleotide polymorphisms (SNPs), which have been identified in genomewide association studies (GWASs). In a meta-analysis of 37,684 people, seven loci were identified to be associated with mean LTL. Five of these loci on genes are involved in telomere biology, including chromosomes 3q26.2 (TERC), −5p15.33 (TERT), 4q32.2 (nuclear assembly factor 1), 10q24.33 (oligonucleotide/oligosaccharide-binding fold containing 1), 18, and 20q13.3 (regulator of telomere elongation helicase 1) [63]. In another meta-analysis of 9190 people, two novel genomic regions that were identified to be associated with LTL variation are near a conserved telomere maintenance complex component 1 (CTC1) on chromosome17p13.1 and zinc finger protein 676 on 19p12 [64].

### 4.3. Cardiovascular Risk Factors

The amount of telomere lost during each cell division varies among people. Previous evidence indicated that increased oxidative stress and chronic inflammation are associated with a higher telomere loss and accelerated telomere shortening [65]. Several common risk factors for CVD [66] such as smoking [67], diabetes mellitus [68], hypercholesterolemia [69], hypertension [70], obesity [71], physical inactivity [72], alcohol consumption [73] and psychosocial problems [74] have been associated with short TL. However, the mechanism underlying the association of telomere shortening with these risk factors remains hypothetical. Most studies have reported that telomere shortening is associated with these risk factors through increased tissue inflammation and oxidative stress [75,76,77]. For example, animal studies demonstrated that hyperglycemia attenuates nitric oxide production in endothelial cells [78], promotes inflammation and oxidative stress [79], and accelerates LTL shortening and vascular atherosclerotic processes [80]. In additional, we found disrupted circadian rhythm results in loss of rhythmic telomerase activities with shortened TL and premature aging in mice. Similar observations also showed in the emergency physicians working in rotating shifts [81].

However, some dietary and lifestyle factors such as marine omega-3 fatty acid [82], antioxidants [23], vitamin intake [83], physical activity [72], and healthy lifestyle [84] were reported to decrease rates of LTL shortening. These factors might contribute to reduced reactive oxygen species, inhibit inflammation, increase endothelial nitric oxide synthase (eNOS) activity, and increased telomerase activity. In an experimental study, voluntary wheel running in mice for three weeks upregulated the activity of telomerase, increased the expression of TRF2, and reduced the expression of vascular apoptosis regulators [85]. However, these exercise-induced changes were absent in both TERT^−/−^ and endothelial nitric oxide synthase (eNOS)^−/−^ mice, indicating the beneficial effects are medicated by TERT and eNOS [29]. A human study also reported that comprehensive lifestyle changes significantly increased telomerase activity and consequently telomere maintenance capacity in human immune system cells [84].

Consequently, telomere shortening is a reflection of cellular aging and a marker of the health status of the aging population [86]. Absolute TL at birth is determined by genetic materials from both parents. During aging, the mean TL declines with cell replication and turnover. The process of telomere shortening is accelerated by exposure to disease-promoting factors such as smoking, obesity, and psychosocial stress. Furthermore, telomerase activation has been considered a possible target for reversing the telomere shortening.

### 4.4. Coronary Artery Diseases

Several studies in diverse populations have reported an association of shorter telomeres in circulating leukocytes with CAD [3,4,5,87,88,89,90,91,92]. The precise mechanisms connecting short telomeres and CAD are yet to be established. Current evidence from epidemiologic and experimental studies supports the role of telomeres in CAD development. First, cardiovascular risk factors such as smoking, hypertension, insulin resistance, and hyperlipidemia, are associated with short LTL. Second, the progression of atherosclerotic plaques in vasculature have been shown to be associated with short TL and cell senescence in vascular cells such as endothelial and vascular smooth muscle cells. Furthermore, short mean LTL represents a greater degree of telomere attrition and senescence in immune cells. Low grade systemic inflammation, which is thought to be mediated by immunosenescence, has been shown to be associated with numerous age-related conditions, including atherosclerosis and cardiovascular diseases.

However, many studies are cross-sectional design and because most cardiovascular risk factors also affect LTL, the causal or consequential relationship between shorter TL and CAD remains controversial. Recently, some prospective longitudinal studies may support the hypothesis that telomere shortening causes CAD, rather than telomere shortening is a consequence of CAD. In a large prospective WOSCOPS study [88], compared with people in the highest tertiles of LTL, those in the lowest tertiles of LTL had a 44% increased risk of coronary artery events in a mean follow-up period of 5.5 years after adjustment for risk factors for CAD. In addition, a recent meta-analysis of prospective studies [91] reported that the estimated relative risk of the shortest versus the longest third of LTL was 1.4 (95% confidence interval: 1.15–1.70). LTL was measured in these prospective trials before the diagnosis of a CVD, thus avoiding the concern of the confounding of reverse causality. Furthermore, reports about the association of genetic variants affecting TL with the risk of CAD also provide evidence for the causal association. The genotypes are randomly determined during conception and thus their associations could be not susceptible to bias and confounding. A meta-analysis of 14 GWASs including up to 22,233 patients with CAD and 64,762 controls revealed that seven SNPs have been identified for the variation in mean LTL. For example, a mean TL decrease of 117 base pairs per TERC telomere-shortening allele accounts for approximately 10% drop in functional telomere reserves in a typical middle-aged adult, and thus increases susceptibility to telomere dysfunction and replicative senescence [93]. The effect of inter-individual variations in LTL is also illustrated in this meta-analysis, which found that the allele associated with shorter LTL increases the risk of CAD; one SD decrease in LTL was estimated to increase the CAD risk by 21% [63].

LTL in patients with CAD has prognostic value. A prospective cohort study of 780 patients conducted for a follow-up period of 4.4 years reported an association of decreased LTL with all-cause mortality, with an adjusted hazard ratio of 1.8 in the lowest TL quartile compared with the highest TL quartile [89]. Moreover, LTL has been observed to be shorter in patients with premature acute MI (aged <50 years) than in healthy, age-matched controls [4]. According analysis in previous studies, patients with MI have TL that is equivalent to that in controls older than 8–12 years [4,91,94]. This might partially explain some young patients with MI without traditional cardiovascular risk factors. Biological aging can reflect the effects of cumulative oxidative stress and inflammatory burden on the aging vasculature. Compared with chronological aging, biological aging may provide superior risk stratification for CVDs. Accurate risk assessment is essential to provide appropriate therapeutic interventions and to further reduce the occurrence of morbid cardiovascular events. New network analysis systems, including genetic traits, imaging characters, and biological risk factors, should be developed for determining the risk of atherosclerosis [95]. We thick LTL could be a sensitive score in the risk prediction system, and additional clinical trials are required to validate the observation and hypothesis.

In the coronary intervention field, researchers observed shorter LTL and increased proinflammatory activity in high-risk unstable plaque (calcified thin-capped fibroatheroma) on virtual histology intravascular ultrasound in patients with acute coronary syndrome also [96]. Furthermore, delayed re-endothelialization after drug-eluting stent (DES) implantation with uncovered stent struts can increase the risk of stent thrombosis. A small clinical trial reported an inverse association of LTL with the percentage of uncovered stent struts, as assessed through optical coherence tomography [97]. Shorter LTL may indicate functional exhaustion and impaired proliferative capacity of EPCs, which are responsible for re-endothelialization after a vascular injury. Additional large-scale prospective studies should be conducted to investigate the clinical application of LTL as a predictive marker for stent thrombosis and target vessel outcomes after DES implantation.

### 4.5. Heart Failure

In a clinical study of 803 patients, LTL was decreased by approximately 40% in patients with HF, and TL in the patients with HF was related to the disease severity [98]. A study investigating the association of a lower left ventricular ejection fraction with decreased TL reported an association of one SD decrease in TL with a 5% lower ejection fraction [99]. Moreover, LTL was significantly associated with cardiovascular outcomes in patients with ischemic HF [100].

HF with a normal ejection fraction was not well recognized until the two previous decades. Approximately half of patients hospitalized for HF have a normal ejection fraction, and outcomes in these patients are equivalent to those in patients with a lower ejection fraction [101]. Aging leads to an increase in the deposition of extracellular matrix components, principally collagen, with an increase in the ratio of type I to type III collagen and a decrease in the elastin content, contributing to impaired ventricular relaxation [102,103]. Furthermore, blunted beta-adrenergic responsiveness, excitation–contraction coupling, and altered calcium-handling proteins contribute to diastolic dysfunction [104]. Studies have reported that the left ventricular relaxation function deteriorates with normal aging and is positively associated with LTL. Older people with shorter LTL have a significantly lower E/A ratio [105].

## 5. Association with Other CVDs

The association of stroke and peripheral artery disease (PAD) [6,21,90,91,92,106,107,108,109,110,111,112] with telomeres has been reported in numerous epidemiological studies. However, the results of these studies are inconsistent and controversial. A prospective survey in a general population of 768 patients for a follow-up period of six years revealed an association of telomere shortening with increased carotid artery intima-media thickness and an increased incidence of cardiovascular events after adjustment for CVD risk factors [111]. However, in a prospective cohort study that included 14,916 healthy American men, no association of relative LTL with ischemic stroke risk was observed [107]. The current available data about PAD are limited and more evidence is required to clarify the association. A cross-sectional study reported an association of LTL with PAD, wherein one SD decrease in LTL significantly increased the risk of PAD by 44% [108]. However, in a prospective longitudinal study, shorter LTL was not associated with an increased incidence of claudication or PAD [92].

Sudden cardiac death (SCD) has been attributed to some genetic variations in the DNA sequence of ion channels. However, it is hypothesized that changes in the gene copy number of potassium (KCNAB1, KCNH2, and KCNA4) and calcium (RyR2, and ATP2A2) channels cause genetic instability and SCD. Moreover, a case-control study reported that patients who experienced SCD had shorter telomeres and changes in the gene copy number of ion channels [113]. This may have been attributed to excessive cellular proliferation caused by oxidative stress stimuli leading to genetic instability in these susceptible people. These findings can provide a new direction to identify a practical marker for predicting SCD risk and making decisions regarding the use of the implantable cardioverter defibrillator device.

In idiopathic pulmonary arterial hypertension, an increase in the proliferation of pulmonary artery smooth muscle cells contributes to the fundamental disease mechanism. A study reported that telomeres in pulmonary arterial smooth muscle cells are longer in patients with idiopathic pulmonary arterial hypertension than in controls and that TL is positively correlated with pulmonary vascular resistance [114]. However, a similar correlation has not been observed in the pulmonary hypertension of other etiologies such as chronic obstructive pulmonary disease.

In patients with degenerative aortic valve stenosis, decreased regenerative capacity and a decreased number of EPCs, caused by cellular senescence, were associated with the progression of degenerative aortic valve stenosis [115].

### Therapeutic Consideration

From the implications of current understanding of telomere biology, potential therapeutic interventions such as the maintenance of TL and modulation of telomerase activity to reverse telomere attrition and cellular senescence, is emerging as a novel strategy for treating atherosclerosis and CVD [50]. Experimental studies have reported that the manipulation of telomerase activity and TL enhances or reverses senescence and aging-associated phenotypes [24,116,117]. For example, telomerase activation therapy after MI successfully prevented ischemic HF in mice. The treatment of adeno-associated viruses with the cardiac-specific telomerase expression resulted in elongated telomeres, attenuated cardiac dilation, improved ventricular function, and smaller infarct scars as well as improved the survival by 17% compared with that of controls [118]. Similar beneficial effects of lifespan expansion with 24% and 13% at one-year and at one-year old mice, respectively and reduction in aging related diseases, without increased cancer susceptibility also reported [119]. However, constitutive expression of telomerase in K5-mTERT transgenic mice revealed an anti-aging effect, accompanying with increased incidence of cancer [120,121]. Furthermore, activation telomerase activity prevent replicative senescence of cardiac fibroblasts results excess extracellularmatrix, fibrosis tissue formation, and pathological remolding after myocardial infarction. Recently published study showed genetic inactivation of premature senescence system resulted in aggravated myocardial fibrosis after transverse aortic constriction in murine model. Conversely, an inducer of premature senescence resulted in 50% reduction of fibrosis [122]. Although the gene transfer therapy targeting telomerase activity has been successful in experimental mice [106,107], several practical limitations must be addressed before its clinical application, especially the concern that the indiscriminate proliferation of telomerized cells might increase the risk of cancer, promotion of myocardial fibrosis, and accelerated atheroma formation due to neointimal VSMC proliferation. However, studies of targeting telomerase therapy in humans are still scarce. A small chemical compound TA-65, extracted from Astragalus membranaceus, is the first described telomere activator. One human study demonstrated dietary supplementation of TA-65 increasing several indicators of health in cardiovascular system and metabolism [123]. Further longitudinal study is mandatory to investigate the anti-aging effects and potential long-term adverse effects. 

Several studies demonstrated the effects of TL maintenance and senescence prevention in certain drugs, which have been used for decades exert clinically beneficial on CVD. For example, statins, 3-hydroxy-3-methylglutaryl-coenzyme A reductase inhibitors, exert various pleiotropic effects to prevent the development of atherosclerotic plaque. They mitigate the genomic damage through potentiation of the DNA repair capacity [124] and upregulation of glutathione synthesis to fight oxidative stress [125]. Furthermore, they can enhance telomerase activity [126] and protect telomere through upregulating TRF2 in endothelial cells and EPCs [127]. A more specific analysis of human T-lymphocytes showed that atorvastatin in pharmacologically relevant doses led to a transient increase in telomerase activity in T-cells. This effect, which could be blocked by inhibitors of Akt and Phosphatidylinositol-4,5-Bisphosphate 3 (PI3)-Kinase, was more pronounced in the CD4-positive (CD4^+^) than in the CD8-positive (CD8^+^) T-cell subset

Angiotensin II has been reported to induce oxidative DNA damage and accelerate cellular senescence in cultured human VSMCs [128]. Therefore, angiotensin-converting enzyme inhibitor or angiotensin II inhibitor can be used to reduce oxidative stress and subsequent DNA damage and senescence. Additionally, pioglitazone, a peroxisome proliferator-activated receptor agonist, can increase the activity of telomerase and expression of TRF-2 as well as reduce the expression of the senescence markers p16, cell-cycle checkpoint kinase 2, and p53 [129].

## 6. Conclusions

Telomere shortening and dysfunction play a crucial role in the pathogenesis of aging-associated CVDs. Critically short telomeres can lead to cellular senescence and apoptosis, which contribute to the development of atherosclerosis and predispose people to plaque instability. Both genetic and environmental factors have been associated with individual variations in TL. Cardiovascular risk factors such as smoking, diabetes mellitus, hypertension, obesity, sedentary lifestyle, and stress have been considered to increase oxidative stress or inflammation, consequently accelerating TL shortening. However, healthy lifestyle and physical activity are protective factors and maintain TL. In clinical practice, shorter LTL reflects the burden of oxidative stress and inflammation, and might be an effective biomarker for risk stratification for atherosclerosis and CVDs. The association of LTL with CAD has been reported in several prospective epidemiological studies, although conclusive evidence of causal relationship is still lacing. However, for subclinical atherosclerosis, ischemic stroke, and PAD, available data are controversial. The role of telomeres in disease pathogenesis should be explored according to some crucial clinical implications of pilot studies, such as coronary artery stenting, SCD, idiopathic pulmonary hypertension, and degenerative aortic stenosis. Furthermore, targeting telomerase or additional telomere-associated proteins may provide a novel therapeutic strategy for neovascularization in patients with ischemic heart diseases and for restoring replicative capacity in those with HF. Additional basic and well-designed clinical studies are required to validate these observations and further expand our knowledge the complexities of telomere dynamics in humans. 

## Abbreviations

The following abbreviations are used in this manuscript:

CVD	cardiovascular diseases
CTC1	conserved telomere maintenance complex component 1
CMV	cytomegalovirus
DES	drug-eluting stent
eNOS	endothelial nitric oxide synthase
EPC	endothelial progenitor cells
GWAS	genomewide association study
HF	heart failure
HSC	hematopoietic stem cells
ICAM-1	intercellular adhesion molecule 1
LTL	leukocyte telomere length
MI	myocardial infarction
PAD	peripheral artery disease
POT1	protection of telomere 1
qPCR	quantitative polymerase chain reaction
Rap1	repressor activator protein 1
ROS	reactive oxygen species
SASP	senescence-associated secretory phenotype
SCD	sudden cardiac death (SCD)
SD	standard deviation
SIPS	stress-induced premature senescence
SNP	single-nucleotide polymorphism
TERC	telomerase RNA component
TERT	telomerase reverse transcriptase
TL	telomere length
TRF1	telomere repeat-binding factor1
TIN2	TRF1 and TRF2 interacting nuclear protein 2
TPP1	tripeptidyl-peptidase 1
VSMC	vascular smooth muscle cells
